# A comparative analysis of the transcriptome profiles of liver and muscle tissue in pigs divergent for feed efficiency

**DOI:** 10.1186/s12864-019-5740-z

**Published:** 2019-06-06

**Authors:** Stafford Vigors, John V. O’Doherty, Kenneth Bryan, Torres Sweeney

**Affiliations:** 1 School of Veterinary Medicine, University College Dublin, Belfield, Dublin 4, Ireland; 2 School of Agriculture & Food Science, University College Dublin, Belfield, Dublin 4, Ireland

**Keywords:** Feed efficiency, Multi-tissue analysis, Pig, Residual feed intake, Transcriptome, RNA-Seq

## Abstract

**Background:**

The improvement of feed efficiency is a key economic goal within the pig production industry. The objective of this study was to examine transcriptomic differences in both the liver and muscle of pigs divergent for feed efficiency, thus improving our understanding of the molecular mechanisms influencing feed efficiency and enabling the identification of candidate biomarkers. Residual feed intake (RFI) was calculated for two populations of pigs from two different farms of origin/genotype. The 6 most efficient (LRFI) and 6 least efficient (HRFI) animals from each population were selected for further analysis of *Longissimus Dorsi* muscle (*n* = 22) and liver (*n* = 23). Transcriptomic data were generated from liver and muscle collected post-slaughter.

**Results:**

The transcriptomic data segregated based on the RFI value of the pig rather than genotype/farm of origin. A total of 6463 genes were identified as being differentially expressed (DE) in muscle, while 964 genes were identified as being DE in liver. Genes that were commonly DE between muscle and liver (*n* = 526) were used for the multi-tissue analysis. These 526 genes were associated with *protein targeting to membrane*, *extracellular matrix organisation* and *immune function*. In the muscle-only analysis, genes associated with *RNA processing*, *protein synthesis* and *energy metabolism* were down regulated in the LRFI animals while in the liver-only analysis, genes associated with *cell signalling* and *lipid homeostasis* were up regulated in the LRFI animals.

**Conclusions:**

Differences in the transcriptome segregated on pig RFI value rather than the genotype/farm of origin. Multi-tissue analysis identified that genes associated with GO terms *protein targeting to membrane*, *extracellular matrix organisation* and a range of terms relating to immune function were over represented in the differentially expressed genes of both liver and muscle.

**Electronic supplementary material:**

The online version of this article (10.1186/s12864-019-5740-z) contains supplementary material, which is available to authorized users.

## Background

Feed is a major economic consideration within the pig industry and accounts for 60–70% of the total production cost. Strategies which improve feed efficiency are a high priority for the pig industry and aim to reduce feed costs and nutrient excretion. Residual feed intake (RFI) is considered the most appropriate measure with which to examine the biological factors contributing to feed efficiency, as differences in production traits such as body weight, growth rate, body composition and maintenance requirements are accounted for within this measure [1]. Significant efforts have been made in recent years to understand the molecular and physiological basis of RFI in pigs and the findings have highlighted a range of processes underpinning this trait, including nutrient digestion and absorption, protein deposition/turnover, energy metabolism and immune function [2–5]. Several studies have identified changes in mitochondrial function as a key contributor to feed efficiency, aligning to their central role in energy metabolism [6, 7]. On a different theme, variation in the immune responses has also been identified as a factor influencing feed efficiency [4, 8, 9].

Irrespective of the underlying biological processes contributing to RFI, early life predictors of feed efficiency would be a valuable, as the measurement of individual feed intake is not feasible in commercial production systems. In this regard, genomic selection is promising as it would allow for early selection without the requirement for direct measurement of feed intake [10]. Enriching SNP panels for functional and biologically important SNPs would improve the accuracy of genomic predictions in a complex trait such as RFI. Feed efficiency has a moderate heritability (0.21–0.33) therefore, genomic selection should facilitate higher rates of genetic gain compared to the traditional selection methodologies based on pedigree and phenotype [11] and this has been explored in pigs in a number of studies [12–14].

Another targeted mechanism of enriching for functional/predictive SNPs is through the selection of SNPs that alter putative transcription factor binding site motifs in the promoter regions of genes that are differentially expressed (DE) between divergent animals. With this approach, it is vital that transcriptomic analysis is performed in tissues with the greatest influence on RFI such as liver and muscle. Genes identified across multiple metabolically relevant tissues and across different populations of pigs will highlight more robust candidate markers for the trait. The liver and muscle are two key metabolically relevant tissues for RFI [6, 15]. In light of this, the objective of this study was to compare the liver and muscle transcriptome of pigs divergent in feed efficiency. Two populations of pigs originating from two different genetic lines and breeding farms were used. We hypothesised that genes associated with energy metabolism and immunity will be identified in the liver and muscle which influence feed efficiency in pigs and will be useful candidates for the identification of functional SNPs.

## Results

### Performance and feed efficiency

Differences in feed intake, performance and feed efficiency traits are presented in Table 1. The animals ranked as LRFI from Farms A and B had a lower RFI value (− 0.14, − 0.18, respectively) compared to the HRFI animals (0.19, 0.20; *P* < 0.001). The LRFI pigs had lower ADFI than pigs ranked as HRFI from both farms (*P* < 0.001). On average, the LRFI group consumed 340 g less feed than their HRFI counterparts in from Farm A and 470 g in Farm B. The MBW, ADG and final BW did not differ between RFI groups (*P* > 0.05).

**Table 1:** Characterisation of performance, body composition, intake and efficiency (Least square means and SEM)

Trait	Low	High^b^	SEM	*P-*value
Farm A	27	18^c^		
RFI	−0.14	0.19	0.08	0.001
ADFI^a^	2.00	2.34	0.04	0.001
MBW	32.71	32.05	0.63	0.956
ADG	0.99	0.99	0.02	0.874
FCR	2.03	2.36	0.02	0.001
BW	76.62	77.70	1.36	0.833
Farm B	28	35		
RFI	−0.18	0.20	0.016	0.001
ADFI^a^	1.87	2.34	0.05	0.0001
MBW	10.94	11.20	0.19	0.3662
ADG	0.950	1.01	0.031	0.2112
BW	90.45	96.08	2.378	0.1086

^a^ *ADFI* Average daily feed intake, *MBW* Mid-test metabolic body weight, *ADG* Average daily gain, *RFI* Residual feed intake, *BW* Body weight

^b^ High = RFI was > 0.5 SD above the mean; medium = RFI was ± 0.5 SD above and below the mean; low = RFI was < −0.5 SD below the mean

^c^ Number of animals in each RFI group

### Transcriptomic differences between RFI groups

**Fig. 1:**
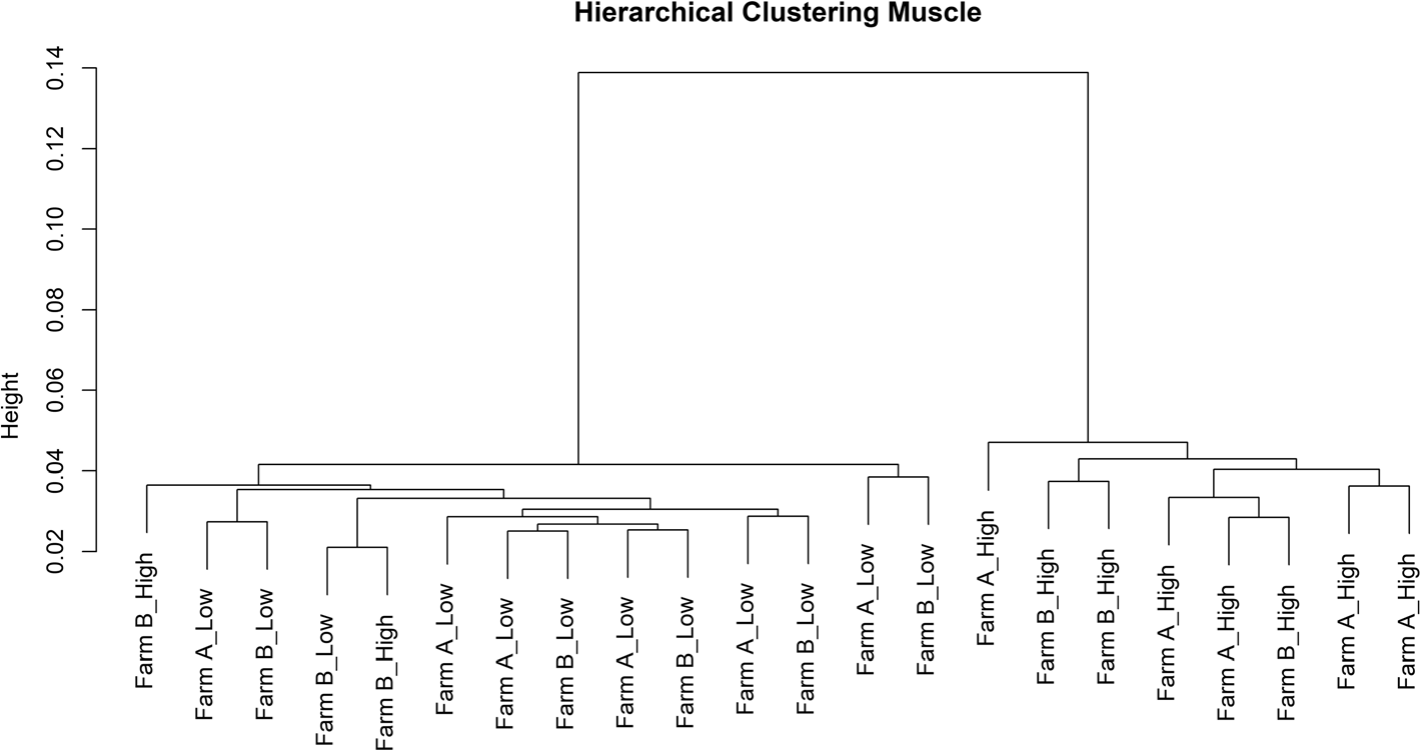
Hierarchical clustering based on transcriptomic differences between the HRFI and LRFI pigs in muscle. The animals grouped by RFI status and not by farm of origin (Farm A and Farm B) in both tissues. The samples were clustered based on Ward method using 1 minus spearman correlation as distance

**Fig. 2:**
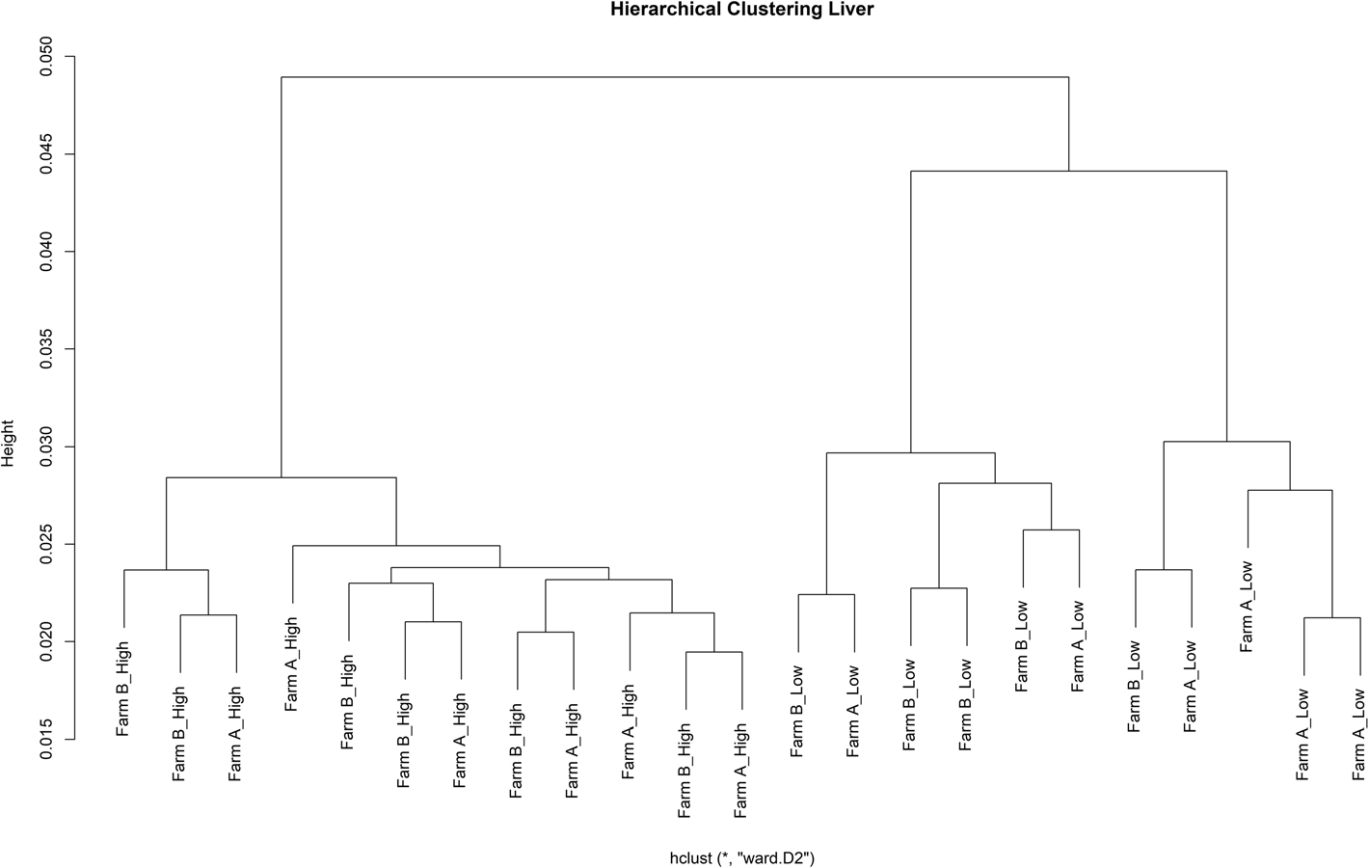
Hierarchical clustering based on transcriptomic differences between the HRFI and LRFI pigs in liver. The animals grouped by RFI status and not by farm of origin (Farm A and Farm B) in both tissues. The samples were clustered based on Ward method using 1 minus spearman correlation as distance

**Fig. 3:**
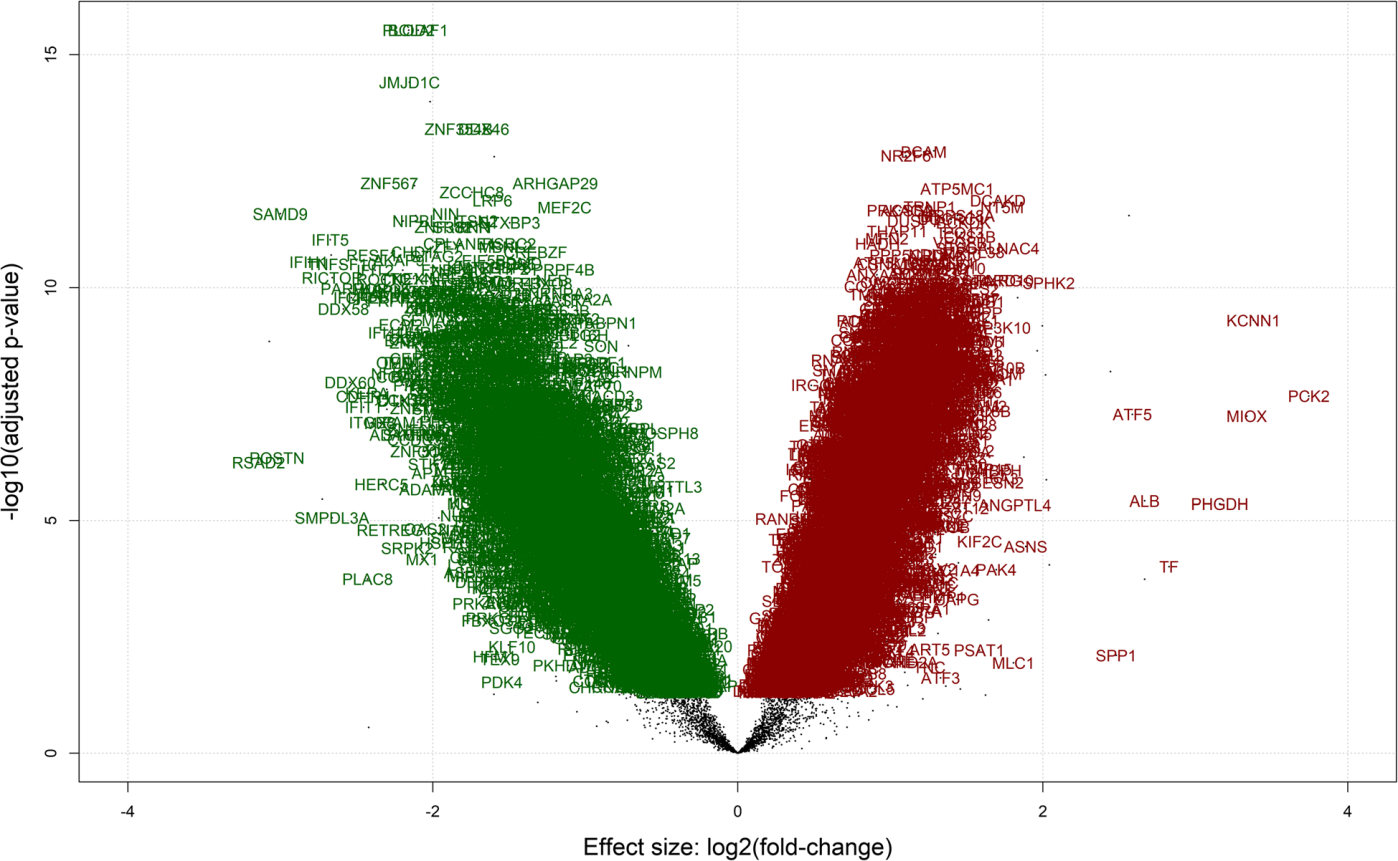
Genes that are differentially expressed between HRFI and LRFI pigs in the muscle (green are up regulated and red are down regulated). Volcano plots illustrate the magnitude and significance of differentially expressed genes between RFI groups

The numbers of raw reads, trimmed reads, and mapped reads for each sample are in Additional file 1: Table S1. As the pigs were obtained from two different farms of origin that used different sires (genotype), hierarchical clustering was performed to establish the impact of the farm of origin on the transcriptome. The hierarchical clustering was performed for both muscle (*n* = 22) and liver (*n* = 23) tissues. The transcriptome for each tissue generally segregated based on the RFI value (Figs. 1 and 2; HRFI & LRFI) rather than genotype/farm of origin. Hence, the data will be presented based on RFI status (i.e. LRFI v HRFI). A total of 6463 genes were identified as being DE in muscle, while 964 genes were identified as being DE in liver (Table 2). Of the 6463 DE genes in muscle, 3138 were up regulated while 3325 were down regulated in the LRFI group (Additional file 2: Table S2). Of the over expressed genes, 1722 had a fold change < 1, 1381 had a fold change between 1 and 2 and 35 had a fold change > 2. In muscle, the volcano plots (Fig. 3) highlight that the fold changes were relatively small. Of the under expressed genes, 2465 had a fold change < 1, 851 had a fold change between 1 and 2 and 10 had a fold change > 2.

**Table 2:** Numbers of differentially expressed genes in LRFI compared to HRFI pigs in both Muscle and Liver

	Muscle^a^		Liver^a^	
Total DE^b^ genes	6463		964	
	Up regulated	Down regulated	Up regulated	Down regulated
Total genes	3138	3325	467	496
FC^c^ < 1.0	1722	2465	365	384
FC > 1.0	1381	851	77	100
FC > 2.0	35	10	25	12

^a^ LRFI vs. HRFI

^b^ Differentially expressed

^c^ Fold change ratio

**Fig. 4:**
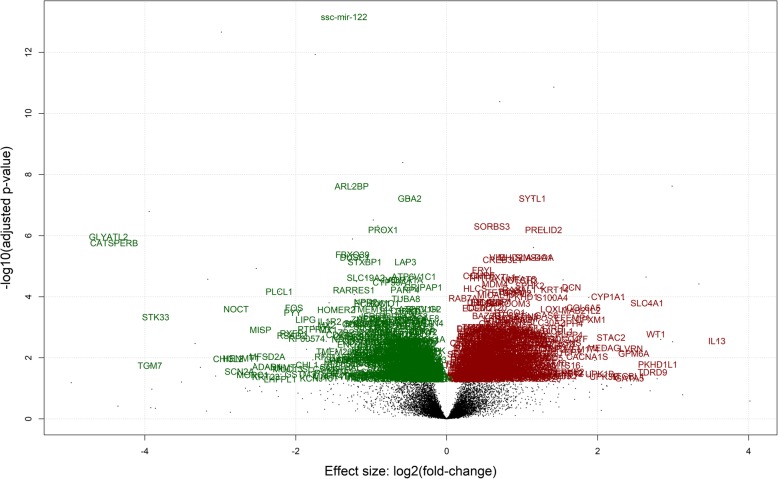
Genes that are differentially expressed between HRFI and LRFI pigs in the liver (green are up regulated and red are down regulated). Volcano plots illustrate the magnitude and significance of differentially expressed genes between RFI groups

In the liver, of the 964 DE genes, 467 were up regulated, while 496 were down regulated in the LRFI group (Additional file 3: Table S3. Of the up regulated genes, 365 had a fold change < 1, 77 had a fold change between 1 and 2 and 25 had a fold change > 2 (Fig. 4). Of the down regulated genes, 384 had a fold change < 1, 100 had a fold change between 1 and 2 and 12 had a fold change > 2.

### Joint analysis of liver and muscle

In both the muscle and liver, of the DE genes, 503 genes (Additional file 4: Table S4). were identified as being commonly DE between the LRFI and HRFI pigs . This is approximately half of the genes DE in the liver.

To aid in the understanding of the biological differences between RFI groups, GO term analysis of DE genes was conducted using a less stringent cutoff i.e. *q*-value < 0.10, thus increasing the range of GO terms. These analyses were conducted separately for both the up regulated and down regulated genes. From the multi-tissue analysis of the DE genes in both muscle and liver, it was observed that the over represented GO terms from the up regulated genes in the LRFI vs. HRFI groups related to immune function and featured the terms*, defense response* and *response to biotic stimulus* (Table 3). The term *defense response* contained the majority of the DE genes (*ADAR, CASP1, CUL1, CXCL10, DDX3X, DHX58, HERC5, IFI27, IFI44L, IFIT1, SGMS1, IFIT2, IFIT3, IFIT5 IFNGR1, IL1RAP, MX1, MX2, NLRP3, NR1D2, OASL, PARP9, PLGRKT, PSME4, RABGEf1, RORA, RSAD2, SP140, TRAF6, TRIM21, XAF1, ZBP1).* In contrast, genes relating to the term’s *protein targeting to membrane* and *extracellular matrix* were under represented in the LRFI vs. HRFI pigs. The term *protein targeting to membrane* contains the genes (*DMTN, GAS6, RPL* (3, 18A), *RPS* (5, 11, 13, 19, 23) and *SSR2)*. The term *extracellular matrix organisation* contains the genes (*CREB3L1, FMOD, GAS6, HTRA1, ITGA3, LTBP2, MMP15, MMP2, NOTCH1 OLFML2B, PDGFA TIMP).*

**Table 3:** Gene ontology terms overrepresented among differentially expressed genes in liver and muscle

Term	Count	B-H *p*-value	
GO terms overrepresented among DEGs with lower expression in the LRFI group vs HRFI group			
GO:0006612~protein targeting to membrane	10	2.59E-02	*DMTN, GAS6, RPL18A, RPL3, RPS5, RPS11, RPS13, RPS18, RPS19, SSR2*
GO:0030198~extracellular matrix organization	12	5.45E-02	*CREB3L1, FMOD, GAS6, HTRA1, ITGA3, LTBP2, MMP15, MMP2, NOTCH1 OLFML2B, PDGFA TIMP*
GO terms overrepresented among DEGs with higher expression in the LRFI group vs HRFI group			
GO:0006952~defense response	32	9.10E-04	*ADAR, CASP1, CUL1, CXCL10, DDX3X, DHX58, HERC5, IFI27, IFI44L, IFIT1,SGMS1, IFIT2, IFIT3, IFIT5 IFNGR1, IL1RAP, MX1, MX2, NLRP3, NR1D2,OASL, PARP9, PLGRKT, PSME4, RABGEf1, RORA, RSAD2, SP140, TRAF6, TRIM21, XAF1, ZBP1,*
GO:0009607~response to biotic stimulus	23	1.21E-03	*ABCA1, ADAR, CASP1, CCDC47, CXCL10, DDX3X, DHX58, HERC5, IFI44L, IFIT1, IFIT2, IFIT3, IFIT5, IFNGR1, MX1, MX2, NLRP3, OASL, RSAD2, TRAF6, CMPK2, FMO1, SGMS1*
GO:0044764~multi-organism cellular process	24	1.42E-03	*ADAR, CXCL10, DDX3X, DHX58, HERC5, IFI44L, IFIT1, IFIT3, IFIT5, IFNGR1, MX1, MX2, NLRP3, NUP153, OASL, PROX1, RSAD2, TOP1, TRIM21, ZMYND11, CFLAR, CUL1, IFIT2, USP7*
GO:0034097~response to cytokine	20	6.75E-03	*ADAR, CXCL10, IFI27, IFIT1, IFIT2, IFIT3, IL1RAP, MX1, MX2, OASL, PARP9, PSME4, RABGEF1, RORA, SGMS1, TRAF6, TRIM21, XAF1, IFNGR1, RSAD2*

### Differentially expressed genes in muscle

Functional analysis using DAVID revealed an over-representation of genes related to the GO terms *cellular protein modification process, RNA metabolic process, gene expression, cell cycle* and *regulation of nitrogen compound metabolic process* with increased expression in the LRFI vs. HRFI groups. In contrast, terms such as *mitochondrion organisation, establishment of protein localisation, cellular amide metabolic process, organonitrogen compound biosynthetic process, macromolecule localisation, cellular localisation, translation* and *generation of precursor metabolites and energy* were over-represented in the down regulated gene list in the muscle of LRFI pigs (Table 4). The genes involved in a selected number of the GO terms are presented in Additional file 5: Table S2. Within the GO term *mitochondrion organisation,* the genes *MFN2, ATG9A, MRPL38, NECTIN2, ATG4D* and *ATP2A1* are most significantly different between the RFI groups. Other genes of interest include: the *MRP* genes (2, 4, 10, 11, 16, 18, 24, 28, 38, 44, 46, 48, 52), the mitochondrial ribosomal genes *MRPS* (2, 6, 24, 26, 34), *NDUF* (A10, AF3, B7, B9, S2, S5, S6, S7, V1,), *NMT1*, *TIMM* (8B, 13, 17B, 22, 34, 40, 44), *UQCC2* and *UQCR10*.

**Table 4:** Gene ontology terms overrepresented among differentially expressed genes in muscle

Term	Count	B-H *p*-value
GO terms overrepresented among DEGs with higher expression in the LRFI group vs HRFI		
GO:0006464~cellular protein modification process	765	1.75E-16
GO:0016070~RNA metabolic process	882	1.05E-12
GO:0010467~gene expression	987	1.05E-12
GO:0007049~cell cycle	371	4.46E-12
GO:0051171~regulation of nitrogen compound metabolic process	831	2.62E-11
GO:0034645~cellular macromolecule biosynthetic process	921	2.43E-10
GO:0006351~transcription, DNA-templated	684	2.94E-09
GO:0070647~protein modification by small protein conjugation or removal	235	2.69E-08
GO:0051276~chromosome organisation	256	5.62E-07
GO:0006396~RNA processing	206	5.83E-07
GO terms overrepresented among DEGs with lower expression in the LRFI group vs HRFI		
GO:0007005~mitochondrion organization	204	1.31E-21
GO:0045184~establishment of protein localisation	445	1.64E-14
GO:0043603~cellular amide metabolic process	241	3.11E-11
GO:1901566~organonitrogen compound biosynthetic process	311	5.75E-11
GO:0033036~macromolecule localisation	562	1.57E-10
GO:0051641~cellular localisation	514	2.00E-10
GO:0006412~translation	167	5.88E-10
GO:0006091~generation of precursor metabolites and energy	112	1.15E-09
GO:0009199~ribonucleoside triphosphate metabolic process	87	4.70E-09
GO:0055114~oxidation-reduction process	228	8.67E-09

### Differentially expressed genes in liver

Functional analysis using DAVID revealed an over-representation of genes with GO terms relating to: *ribonucleoprotein complex biogenesis*, *RNA processing*, *defense response to virus, type 1 interferon signaling pathway*, *response to cytokine*, *RNA metabolic process*, *carboxylic catabolic process, defense response*, *lipid homeostasis* and *positive regulation of gene expression* in the genes up regulated in the LRFI pigs (Table 5). The terms *cell surface receptor signaling pathway*, *extracellular matrix organisation*, *extracellular structure organisation,* *biological adhesion*, *cell adhesion*, *regulation of multicellular organismal development*, were down regulated in LRFI pigs*.* The genes involved in a selected number of the GO terms are presented in Additional file 6: Table S6.

**Table 5:** Gene ontology terms overrepresented among differentially expressed genes in liver

Term	Count	B-H *p*-values
GO terms overrepresented among DEGs with higher expression in the LRFI group vs HRFI group		
GO:0022613~ribonucleoprotein complex biogenesis	35	2.25E-04
GO:0006396~RNA processing	53	2.29E-04
GO:0051607~defense response to virus	22	5.93E-04
GO:0060337~type I interferon signalling pathway	13	5.99E-04
GO:0034097~response to cytokine	45	0.001367
GO:0016070~RNA metabolic process	161	0.004935
GO:0046395~carboxylic acid catabolic process	18	0.005979
GO:0006952~defense response	67	5.93E-04
GO:0055088~lipid homeostasis	14	0.007424
GO:0010628~positive regulation of gene expression	71	0.012472
GO terms overrepresented among DEGs with lower expression in the LRFI group vs HRFI group		
GO:0007166~cell surface receptor signalling pathway	120	1.86E-06
GO:0030198~extracellular matrix organisation	32	1.91E-06
GO:0043062~extracellular structure organisation	32	1.37E-06
GO:0022610~biological adhesion	85	4.48E-06
GO:0007155~cell adhesion	84	6.60E-06
GO:2000026~regulation of multicellular organismal development	78	6.00E-04

## Discussion

The current analysis provides novel findings in relation to the genes and processes associated with feed efficiency in pigs. We hypothesised, based on previous research, that genes associated with energy metabolism and immunity would be key influencers of feed efficiency. The results from this study partially support this hypothesis. In the joint analysis, variation in the expression of genes with the GO terms *protein targeting to membrane*, *extracellular matrix organisation* and a range of terms relating to immune function were DE between the LRFI and HRFI pigs. The analysis of the muscle tissue alone, highlighted genes with GO terms relating to RNA, protein synthesis and energy metabolism. The analysis of the liver tissue alone, highlighted genes with GO terms involved in *lipid homeostasis*. This analysis identifies a number of genes that are DE in both tissues and others which are tissue specific, all of which may be influencing RFI.

### Differentially expressed genes

There was a notable difference in the number of DE genes in the muscle and liver with a 10-fold increase in genes expressed in the muscle compared to the liver. Interestingly, many of the GO terms in the muscle related to RNA and protein synthesis, supporting the increased transcriptomic activity in this tissue. A high proportion of DE genes had relatively subtle changes in gene expression with fold changes < 1. This is consistent with other transcriptomic studies assessing variation in RFI in pigs, with the majority exhibiting small fold changes [7, 16–18]. While the reasons for the small fold changes in gene expression are unclear, Liu et al. [15] postulated that as pigs were not exposed to any external stimulus/challenge and were healthy, these expression levels therefore reflect the normal physiological range.

### Combined analysis of muscle and liver

For the purposes of identifying robust markers of feed efficiency, we propose that the genes DE between LRFI and HRFI pigs in both muscle and liver are the most biologically significant. Indeed, 503 genes were commonly DE in the muscle and liver between the LRFI and HRFI pigs. Interestingly, this constituted approximately half of the genes expressed in the liver. The genes mainly grouped with three functions: *protein targeting to membrane, extracellular matrix* and *immune function*. A range of biologically relevant genes were DE within the term *protein targeting to membrane* (*GAS6*, *RPL* (19, 39), *RPS* (5, 11, 13, 18, 19) and *SSR2). GAS6* had lower expression in the LRFI group in comparison to the HRFI group. *GAS6* which binds to the TYR03 receptor has previously been associated with cell differentiation in adipocytes and obesity in both humans and mice [19]. Interestingly in muscle, *TYRO3* was DE between LRFI and HRFI pigs in the same direction to *GAS6*. It has previously been shown that LRFI pigs are leaner and have greater muscle content than their HRFI counterparts [18, 20, 21]. The changes in *GAS6* and *TYRO3* expression may influence body fat and increase efficiency in LRFI pigs. The majority of other genes in this term (*RPL* (3, 18A), *RPS* (5, 11, 13, 18, 19) and *SSR2)* are associated with ribosomal biogenesis and protein translation, functions which are important for cell growth and proliferation [22] and also the regulation of skeletal muscle mass [23]. All these genes had lower expression in the LRFI group in comparison to the HRFI group. Interestingly, an upstream regulator of ribosomal biogenesis, *mTOR,* had lower expression in the LRFI group in comparison to the HRFI group. mTOR has previously been attributed to the control of ribosomal biogenesis, a potential driver of the DE of the *RPL* and *RPS* genes and a factor impacting on differences in efficiency [23]. It could be inferred that the reduced expression of ribosomal proteins could contribute to energy reduction in the LRFI pigs.

The over-represented GO terms derived from the genes up regulated in the LRFI vs HRFI groups related to immune function and featured the terms*, defence response* and *response to biotic stimulus*. It was initially hypothesised that more efficient animals may be more susceptible to disease and stressors [24]. However, the more efficient LRFI pigs have been found to be more robust and better able to respond to infectious challenge [8]. With regard to RFI, immune-related data is highly variable and conflicting across transcriptomic studies. In some studies of both more efficient LRFI pigs and cattle there was also an up regulation of immune related genes [25, 26]. However, more efficient pigs generally have a lower expression of genes related to immune function in non-challenged states [16, 17, 27], an observation that is highly tissue dependent. The studies performed by Jégou et al [27] and Liu et al [16] differed from the current study, both in their findings and context, as the blood transcriptome, rather than that of the liver and muscle, was studied. However, the differing findings of Gondret et al [17] which included the analysis of muscle and liver, are more challenging to interpret. One potential explanation is that the pigs in this study were subjected to a pathogen/viral challenge in the form of vaccination or infection and hence a heightened immune response is reflected in this cohort. The pigs in this study received vaccination against post weaning multi-systemic wasting syndrome (PMWS). Potentially, the vaccination program elicited an immune response in early life which persisted to slaughter, thus up regulating immune related genes and subsequently altering the ranking of the DE genes. Previously, a gene expression profile associated with viral immunity in adult sheep was attributed to vaccination in early life [28]. Interestingly, the expression pattern of several members of the gene families adenylate synthetase-like protein (OAS) and interferon-induced protein (IFIT) were common between the two studies. The fact that all animals were healthy prior to slaughter based on performance records, suggests that the increased expression of immune related genes did not have any negative effects on the animals in this study.

#### Muscle

A range of GO terms involved with RNA and protein synthesis had higher expression in the LRFI pigs compared to the HRFI group in muscle, with the most significant term being *cellular protein modification process*. This term is associated with the post translational modification of amino acids. The term relates to a number of genes (*PRKGC1*, *MSTN*, *MAP3K1*, *ZNF451, IGF1*) all of which could potentially be key to the differences in efficiency between RFI groups. In particular, the gene *IGF1* has greater expression in the LRFI group compared to the HRFI pigs. Previously, *IGF1* has been shown to correlate with RFI [29]. IGF1 is known to influence growth and development and therefore, could potentially be a key driver of feed efficiency [30]. Previously, several micro-RNAs were up regulated in LRFI pigs which the authors attributed to increased muscle growth and development through the IGF1/mTOR signalling pathway. Another gene of interest within this GO term is *MSTN* due to its functional role in muscle growth and development [31]. Similarly, *MAP3K1* expression has been reported to be involved in tissue formation through protein synthesis [32]. The effect of changes in the expression of genes associated with muscle development in animals that have similar growth warrant further exploration. As the LRFI and HRFI pigs did not differ in ADG by having an increase in genes such as *MAP3K1* these pigs may be more efficient at laying down muscle. Previously, genes associated with the term *gene expression* were identified as being significantly up regulated in adipose tissue, which is in agreement with the up regulation of genes related to this term in the liver of LRFI pigs [33]. Of the highlighted GO terms, the term *translation elongation,* a child term of *translation* was previously identified as being down regulated in LRFI pigs across a range of tissues, including liver, muscle perirenal and subcutaneous adipose tissue [17].

In addition, the GO term, *mitochondrion organisation* was most significantly down regulated in the LRFI compared to their HRFI counterparts in muscle. In combination with the GO term *mitochondrion organisation*, the terms *generation of precursor metabolites and energy* and *oxidation reduction processes* are terms which contain genes associated with mitochondrial function/energy metabolism. Mitochondria produce 95% of energy within eukaryotic cells [34]. Changes in the expression of genes involved in the control of mitochondrial function are potentially key drivers of alterations in feed efficiency in pigs. Jing et al. [7] proposed that the LRFI pigs may be more efficient either as a result of reduced numbers of mitochondria or a down regulation of mitochondrial function. A number of genes; *COX, NDUF, SLC*, and *UQCR* were up regulated in the HRFI compared to the LRFI pigs in this study, which is in agreement with previous studies [6, 7, 33, 35]. *PPARGC1B* was also down regulated in the LRFI pigs in this study. *PPARGC1B* is a master regulator of mitochondrial biogenesis; the down regulation of this gene may be a key driver of differences in efficiency between LRFI and HRFI pigs. Increased expression of *PGC* gene families such as *PPARGC1B* has been reported to increase energy expenditure and mitochondrial number [36]. The results suggest that ATP synthesis is potentially lower in the LRFI pigs than the HRFI pigs.

#### Liver

Genes involved in the GO term *cell surface receptor signaling pathway* were the most significantly up regulated genes in the liver of LRFI pigs relative to the HRFI group. This is a very broad GO term that has previously been identified as over-represented in the blood transcriptome of pigs from divergent selection lines [15]. The most significantly DE genes were *VIM*, *CCNY*, *OGN*, *DCN* and *KRT19*. These genes have a range of functions, including cell division, cell structure and bone formation. Genes associated with appetite control/feed intake*/*energy metabolism *APOA4, LEAP2, RORA, PPARα* were identified within the GO term *defense response*. *APOA4* was up regulated in the LRFI pigs, which was also observed in a different population of LRFI pigs with a similar genetic background to the pigs from Farm A in this study [17]. *APOA4* has a role in satiety, is involved in the inhibition of appetite and has been attributed to the long-term regulation of feed intake [37, 38]. Similarly, *LEAP2, RORA* and *PPARα* were up regulated in the LRFI pigs. LEAP2 is an antagonist of the growth hormone secretagogue receptor 1a that binds to ghrelin, and is a key factor regulating systemic energy metabolism [39]. RORA is an upstream regulator of the *PPARα/RXRA* complex. This complex acts as a transcription factor that activates genes involved in glycolysis/gluconeogenesis [40]. *PPARα* has previously been associated with feed efficiency in pigs [33]. These genes are of interest as more efficient (LRFI) pigs have lower feed intake than HRFI pigs, suggesting differences in appetite regulation and energy metabolism [33, 41].

Genes involved in the GO term *lipid homeostasis* were over-expressed in the liver of LRFI pigs compared to the HRFI animals. Interesting up regulated genes associated with this term included *LIPG*, *ACADL*, *ALSM1* and *APOA4.* LIPG plays a role in lipoprotein uptake and metabolism [42] and *ACADL* is involved in the beta-oxidation of fatty acids. *ALSM1* is also involved in adipogenesis [43]. Lipogenesis in the liver generates fatty acids, which are esterified into triglycerides for storage in adipose tissue, oxidized in the liver or exported to other parts of the body as lipoproteins where they are used as an energy source and as membrane building components [44]. Based on this statement the increased expression of the genes associated with this GO term suggest that the more efficient (LRFI) pigs have improved capacity to metabolise fats, which may be an important driver of the improved efficiency in this group.

## Conclusion

Overall the transcriptome data segregated based on RFI value rather than the genotype/farm of origin. Genes associated with GO terms *protein targeting to membrane*, *extracellular matrix organisation* and a range of terms relating to immune function were over represented in the DE genes of both liver and muscle. In the muscle, genes associated with GO terms involved in RNA, protein synthesis and energy metabolism were DE between efficient and inefficient pigs. In the liver, genes associated with *lipid homeostasis* were DE between the RFI groups. This suggests that there are common pathways across tissues as well as tissue specific pathways that contribute to differences in feed efficiency. These results highlight key genes *APOA4, CPT1A, GAS6, IGF1, LEAP2, MAP3K1, MDUFB9, MSTN, mTOR, PPARα, PPARGC1B, PRKGC1, RORA, TYR03* and *ZNF451* as potential candidates for the identification of functional SNPs.

## Methods

All experimental procedures described in this work were approved under University College Dublin animal research ethics committee (AREC-15-30-O’Doherty) and conducted under experimental license from the Department of Health in accordance with the cruelty to animal act 1876 and the European Communities (Amendments of Cruelty to Animal Act, 1876) Regulations (1994).

Two populations of pigs (Farms A and B) were utilised in this study. Pigs divergent in RFI were evaluated using a standard method [2]. Experimental conditions remained consistent between the two populations of pigs. The first population of pigs were the progeny of sows (Landrace x Large White) bred to Meatline boars (Farm A: Maxgro, Hermitage Pedigree Pigs, County Kilkenny Republic of Ireland) while the second population of pigs were progeny from sows (Landrace x Large White) bred to Meatline boars (Farm B: PIC Genetics, Line 37) and originated from a different breeding farm. The pigs in this study were vaccinated against post-weaning multi-systemic wasting syndrome (PMWS) and received the vaccination (CIRCOVAC) prior to weaning.

At weaning (day 28), pigs (144 and 140 pigs from Farms A and B, respectively) were transferred from their respective farms to UCD Lyons Research Farm (Newcastle, Co. Dublin, Ireland) and reared on standard commercial diets until slaughter and grouped in pens. Feed intake was recorded using single space computerized electronic feeders (Mastleistrungsprufing MLP-RAP; Schauer Agrotronic AG, Sursee, Switzerland) according to the method of Varley et al [45]. To calculate RFI in population A, the BW of each animal was recorded on day 56 and subsequently on days 63, 70, 77, 84, 91, 98 and 105. Day 77 BW was used to calculate midtest metabolic BW (MBW). On day 105, a backfat (BF) scan was taken from all pigs in the area over the 10th rib. To calculate RFI in population B*,* the body weight of each animal was recorded on day 92, and subsequently on day 99, 106, 113, 120, 127, 134 and 141. On day 133, bodyweight was used to calculate the mid-test metabolic body weight (MBW). On day 133, a backfat (BF) scan was taken from all pigs in the area over the 10th rib. In both populations, mid-test metabolic body weight was included and represented as BW^0.60^ which is representative of the maintenance requirement of the animal [46, 47]. The RFI was the residual calculated using multiple regression model regressing average daily feed intake (ADFI) to average daily gain (ADG), BF and MBW. Standard deviations above and below the mean were used to group animals into HRFI (RFI > 0.5 SD above the mean), and LRFI (RFI < − 0.5 SD).

### Animal selection and slaughter procedure

In both Farms A and B, the 6 most efficient (LRFI) and 6 least efficient (HRFI) animals were selected for further analysis. Following fasting for a three-hour time period, the pigs were euthanized by lethal injection with Euthatal (Pentobarbitone Sodium BP; Merial Animal Limited) at a rate of 1 ml/2 kg body weight. Following slaughter, tissue sections (1cm^2^) were sampled from the liver and *Longissimus Dorsi* muscle and stored in RNA*later* (Ambion Inc., Austin, TX). Following an overnight incubation at 4 °C, the RNA*later* was removed and tissue was stored at -70 °C until RNA extraction.

### RNA extraction

Trizol Reagent (Sigma-Aldrich, Arklow, Ireland) was used to extract total RNA from liver (50 mg) and muscle (100 mg). The GenElute Mammalian Total RNA Miniprep Kit (RTN70, Sigma-Aldrich) was used to further purify the crude RNA extract and included an on-column DNase step. The NanoDrop-ND1000 Spectrophotometer (Thermo Fisher Scientific Inc. MA, USA) was used to quantify RNA using the ratio of the absorbance at 260 and 280 nm with ratio above 1.8 being the acceptable cut-off. The Agilent RNA 6000 Nanochip bioanalyzer kit was used to assess RNA integrity with all samples having an RNA Integrity Number (RIN) above 8 (8.3 s.e. 0.59).

### RNA-sequencing

The Institute of Molecular Medicine, University of Leeds, United Kingdom performed the library construction and RNA-seq. The RNA-seq libraries were constructed using the Illumina TruSeq RNA Sample Preparation Kit v2 (Illumina, San Diego, CA) according to the manufacturer’s instructions. Adapters with unique barcodes were ligated to the end-polished cDNA fragments for each sample. The libraries were amplified by PCR, size selected and quantitated. The individual libraries were pooled with 24 libraries per pool. One hundred base paired-end sequencing was run on an Illumina HiSeq2000 platform with each pool ran on two lanes on a flow cell.

### Quality control read processing and alignment

Read quality was checked by using FastQC (0.11.5). Adapters and low-quality reads were trimmed using the program Cutadapt (1.14) such that the average base quality was greater than 20. Trimmed paired end reads were aligned to the pig reference genome Sscrofa 10.2 (version 87, Ensembl) using the alignment program HISAT2 (version 2.0.5), using the default settings [48, 49]. SAM files were sorted using the sort procedure of SAMtools [50, 51]. Using the program FeatureCounts (1.5.2), the sorted SAM files were used to generate read counts expressed per gene per library while the pig genome GTF file (version 10.2.87, Ensembl) as used as the genomic reference annotation file and default settings were applied [52].

### Statistical analysis of animal performance

The performance data for both populations was analysed using the MIXED procedure (SAS Inst (version 9.4). Inc., Cary, NC) as a complete randomised design with RFI as the main effect and sow included as a random effect. Each population of animals was analysed separately. For all parameters examined, the individual pig was the experimental unit. The data was checked for normality using the UNIVARIATE procedure of SAS. All data presented in the tables are expressed as least squares means ± standard error of the mean (SEM). Means were separated using the Tukey-Kramer method. The probability value, which denotes statistical significance was *P* < 0.05.

### Statistical analysis of gene expression data

The statistical language R was used for all statistical analyses of RNA-seq data (version 3.4.0). Count files produced from FeatureCounts were merged using the readDGE function within the R package edgeR (version 3.18.1) to produce a count table [53, 54]. Hierarchical clustering was applied to identify outliers and based on this, one sample was removed from the liver dataset while two samples were excluded from the muscle data. Differential gene expression analysis was conducted using functions within the R package DESeq2 (version 1.16.1) [55]. As both populations grouped based on RFI, the gene expression data from Farms A and B were analysed together. The gene count data was normalised using the relative log expression method, based on “size factors” which accounts for RNA-seq library size differences. Dispersion estimates were also calculated. Pairwise comparisons of expression were then made between the LRFI and HRFI groups for each gene based on a negative binomial regression with farm included in the model. The output from DESeq2 included fold changes and associated *p*-values and adjusted *p*-values. A gene was defined as differentially expressed (DE) if the Benjamini Hochberg adjusted *p*-value was < 0.05. To account for variation between the populations, the farm of origin was included in the model. To facilitate further analysis, porcine Ensembl IDs were first uniquely mapped (one to one relationship only) to the more extensively annotated human orthologs, retrieved from the BioMart database [56]. All subsequent analyses were performed using these human orthologs and annotations.

The lists of DE genes were investigated for enrichment analysis of gene ontology (GO) terms for Biological Processes (BP), using the functional annotation tool of Database for Annotation, Visualization and Integrated Discovery (DAVID) bioinformatics resources [57]. The gene lists input in to DAVID were divided into over and under-represented groups in both liver and muscle. The *background* gene list used in the over and under-representation analysis included all genes with a one-to-one mapping with human orthologs for both liver and muscle. The GO term GOTERM_BP_FAT was selected to filter GO terms. The GOTERM_BP_FAT filters out broad GO terms based on the measured specificity of each term. For functional annotation analysis, GO terms were considered to be significant if they had a *q*-value of (*q* < 0.10). To identify commonalities between liver and muscle, the VENNY online tool [58] was used to compare the gene lists for up and down regulated genes in both tissues.

## Additional files

Additional file 1: **Table S1.** Sequencing information. This file contains raw sequence, read alignment and gene annotation information. (XLSX 13 kb)

Additional file 2: **Table S2.** Differentially expressed genes in muscle. This table lists the genes that were differentially expressed in muscle. (XLSX 705 kb)

Additional file 3. **Table S3.** Differentially expressed genes in liver. This table lists the genes that were differentially expressed in liver. (XLSX 118 kb)

Additional file 4: **Table S4.** Common differentially expressed genes in liver & muscle. This table lists the genes that were differentially expressed in both liver and muscle. (XLSX 27 kb)

Additional file 5: **Table S5.** Selected gene ontology terms in muscle. This table lists the genes involved in a selected number of gene ontology terms identified in muscle. (DOCX 23 kb)

Additional file 6: **Table S6.** Selected gene ontology terms in liver. This table lists the genes involved in a selected number of gene ontology terms identified in liver. (DOCX 16 kb)

## Abbreviations

DAVID: Database for Annotation, Visualization and Integrated Discovery
GO: Gene ontology
HRFI: High residual feed intake
LRFI: Low residual feed intake
RFI: Residual feed intake
