# Supplementary material for: A comparative analysis of the transcriptome profiles of liver and muscle tissue in pigs divergent for feed efficiency
Source: BMC Genomics. 2019 Jun 6;20:461. doi: 10.1186/s12864-019-5740-z (PMC6555042; doi:10.1186/s12864-019-5740-z)
Supplement: Supplementary file 5 — Table S5. Selected gene ontology terms in muscle. This table lists the genes involved in a selected number of gene ontology terms identified in muscle. (DOCX 23 kb) [file 12864_2019_5740_MOESM5_ESM.docx]

| ***GO:0006464~cellular protein modification process (overrepresented among DEGs with higher expression in the LRFI group vs HRFI)*** |
| --- |
| *ABCA1 ABL2 ABRAXAS2 ACER2 ACTL6A ADAM10 ADAM9 ADAMTS5 ADAR ADCY4 ADNP AGBL3 AGTPBP1 AKAP8 AKT3 ALG6 ALPK1 ANGPT1 ANKIB1 ANKRD6 ANXA1 APC APIP ARFGEF1 ARID4A ARID4B ARIH1 ARL6IP5 ARMT1 ARPP19 ARRDC3 ARRDC4 ARSJ ARSK ART3 ART4 ASB15 ASB4 ASB5 ASB7 ASPN ATE1 ATF2 ATG12 ATG14 ATG3 ATG4C ATP7A ATR ATRX ATXN3L ATXN7 B3GALNT1 B3GALT2 B3GAT2 B3GNT5 B4GALNT2 BARD1 BAZ1B BAZ2A BCCIP BIRC2 BIRC3 BIRC6 BLM BMP4 BMP5 BMPR1A BMPR2 BNIP2 BRAF BRCA1 BRCC3 BRMS1L BTBD1 BTBD3 C3orf33 CAMKK2 CAPRIN2 CASK CAV1 CAV2 CBLB CCDC126 CCDC88A CCNA2 CCNC CCNE2 CCNG1 CCNH CCNL1 CCNT1 CCR5 CD109 CD24 CD80 CDC14A CDC14B CDC20 CDC25C CDC27 CDC42BPA CDC42BPB CDC7 CDC73 CDCA2 CDH2 CDK12 CDK13 CDK14 CDK17 CDK7 CDK8 CDKL1 CDKL3 CDKL5 CDKN3 CENPE CHEK1 CHM CHML CHORDC1 CHUK CLK4 CLOCK CNOT4 CNTN1 COG3 COL4A3BP COPS2 COPS5 CPNE3 CRBN CREB1 CRY1 CSNK1G3 CTDSPL CTH CTR9 CTTNBP2NL CUL1 CUL2 CUL5 CWC27 CXCL10 CYLD CYSLTR2 DAB2 DAPP1 DCAF1 DCAF10 DCAF13 DCAF17 DCN DCUN1D1 DCX DDR2 DEPTOR DLG1 DLGAP5 DNAJA1 DNAJC10 DNAJC24 DNAJC3 DNAJC6 DOCK7 DPM1 DPY19L1 DPY19L3 DPY19L4 DR1 DSTYK DTX3L DUSP11 DUSP4 DYRK3 DZIP3 EDEM1 EDEM3 EDNRB EFEMP1 EGF EHHADH EID1 EIF2A EIF2AK3 EIF2S1 EIF3A ELK4 ELOC ELP4 ENPP1 ENTPD5 EOGT EPC1 EPHA3 EPHA4 EPHA5 EPHA7 ERCC6 ERCC8 ERG ERO1A ESCO1 ESCO2 ETF1 EZH1 EZH2 F2R FAM129A FAM76A FAM76B FAS FASTKD1 FBN1 FBXL3 FBXL4 FBXL5 FBXO11 FBXO22 FBXO24 FBXO30 FBXO32 FBXO40 FBXO5 FEM1B FEM1C FER FGF10 FGF12 FGF2 FGF9 FGFR1OP2 FKBP14 FKBP3 FKBP5 FKBP7 FKTN FLRT3 FMR1 FRS2 FUT8 FYB1 FZD4 G2E3 GALNT1 GALNT5 GATA2 GCLC GCNT1 GCNT4 GHR GLMN GNL3 GRK7 GTF2H1 GTF2H3 GXYLT2 HACD3 HACE1 HAT1 HDAC2 HECTD1 HECTD2 HECW1 HECW2 HERC1 HERC4 HERC5 HGF HIPK3 HLCS HLTF HMGCR HS3ST5 HSF4 HSPH1 IBTK ICK IGF1 IL15 IL17RD IL2RA IL6ST IMPACT ING2 ING3 INPP5F IRAK4 ITGA1 ITGAV JADE1 JADE2 JADE3 JAK2 JMJD1C KANSL1 KANSL1L KAT2B KAT6A KAT6B KBTBD12 KBTBD2 KBTBD3 KBTBD8 KDM1B KDM3A KDM6A KDM7A KIT KITLG KLF4 KLHL15 KLHL20 KLHL23 KLHL24 KLHL28 KLHL31 KLHL4 KLHL42 KLHL5 KLHL7 KLHL8 KLHL9 KMT2A KMT2E KMT5A KRAS LATS1 LCP2 LEO1 LIAS LIMK2 LMAN1 LMO7 LNPEP LNX1 LONRF1 LOX LPAR1 LRP6 LRR1 LRRC66 LRRK2 LTN1 MAGI3 MAGT1 MALT1 MAML1 MAN1A1 MAN1A2 MAN2A1 MAP3K1 MAP3K15 MAP3K2 MAP3K20 MAP4K3 MAPK6 MAPK8 MAPK9 38412 39142 MBIP MCFD2 MDFIC MDM2 MDM4 MECOM MED1 MEF2C MELK METAP2 METTL11B MGAT2 MGAT4A MIB1 MINDY2 MINDY3 MITF MLKL MOB1B MORC3 MORF4L2 MPHOSPH8 MRE11 MSL2 MSTN MTF2 MTM1 MUSK MYCBP2 MYSM1 N4BP1 NAA15 NAA16 NAA25 NAA35 NAA50 NBN NCK1 NCOA1 NCOA3 NDFIP2 NDUFAF7 NEDD4 NEK1 NEK3 NEK4 NEK7 NF1 NFE2L2 NGLY1 NIPBL NKTR NMI NMT2 NOP58 NOX4 NPM1 NPTN NR2C2 NRP1 NSD3 NSMCE4A NUB1 NUP133 NUP153 NUP155 NUPL2 NUS1 OAS2 OGA OGT OSBPL8 OTUD4 OXTR P4HA1 P4HA3 PABPN1 PAFAH1B1 PAK2 PAM PAN3 PARP2 PAXBP1 PBLD PCMTD1 PCNP PDCD10 PDE4D PDE5A PDE8A PDGFD PDIK1L PDK1 PDK4 PDP1 PELI2 PGGT1B PHC3 PHF20 PHF21A PHLPP1 PIGA PIGM PIGN PIK3C3 PIK3CA PIK3R4 PIN4 PJA2 PKD2 PKIA PKN2 PLCB1 PLCE1 PLCG1 PLCL1 PLK2 PLK4 PLOD2 PLPP3 PODN PPID PPIG PPIL3 PPIL4 PPM1A PPM1B PPP1CB PPP1CC PPP1R12A PPP1R15B PPP1R9A PPP2R2A PPP2R5A PPP3CA PPP4R3B PPP6R3 PRDM5 PRKACB PRKAG2 PRKCG PRKCH PRKCI PRKD3 PRKDC PRKG1 PRKX PRMT3 PRMT9 PRNP PROS1 PROX1 PRPF4B PSMA3 PSMC6 PSMD12 PSMD14 PSME4 PTAR1 PTEN PTP4A2 PTPN1 PTPN11 PTPN12 PTPN13 PTPN2 PTPN4 PTPRA PTPRB* |
| **GO:0007005~mitochondrion organization (*overrepresented among DEGs with lower expression in the LRFI group vs HRFI)*** |
| *AARS2 ACAD9 AFG3L2 AIFM1 AIFM2 AIP ALAS1 ALKBH7 AMBRA1 APOO ARIH2 ARRB2 ATG2A ATG4D ATG7 ATG9A ATP2A1 ATP5F1B ATP5IF1 BAD BAK1 BAP1 BCL2L1 BID BNIP3 C19orf70 CCAR2 CDK5RAP1 CHCHD1 CHCHD10 CHCHD2 CHCHD4 CLEC16A CLUH CNP COA3 COX17 CYB5R1 DNAJA3 DNM2 DYNLL1 DYNLL2 E2F1 EARS2 ECSIT EIF4G1 ELAC2 EPAS1 ESRRA FAM162A FOXRED1 FUNDC2 FXN FZD9 GABARAPL2 GADD45GIP1 GBA GPER1 HARS HCFC1 HDAC6 HIP1R HMGCL HPS4 HSD17B10 HTT IMMP1L IMMT JTB KAT2A LETM1 LMNA MAP1LC3A MARK2 MFN2 MIEF1 MIGA2 MPV17L2 MRPL10 MRPL11 MRPL14 MRPL15 MRPL16 MRPL17 MRPL18 MRPL2 MRPL20 MRPL21 MRPL24 MRPL28 MRPL36 MRPL38 MRPL4 MRPL40 MRPL43 MRPL44 MRPL46 MRPL48 MRPL49 MRPL52 MRPL54 MRPS11 MRPS14 MRPS2 MRPS24 MRPS25 MRPS26 MRPS27 MRPS34 MRPS6 MRPS7 MRRF MTCH2 MTERF4 MTFP1 MTFR1L MTX1 MUL1 MYH14 NAIF1 NDUFA1 NDUFA10 NDUFA13 NDUFA8 NDUFAB1 NDUFAF3 NDUFB1 NDUFB11 NDUFB2 NDUFB6 NDUFB7 NDUFB9 NDUFS2 NDUFS3 NDUFS5 NDUFS6 NDUFS7 NDUFV1 NDUFV2 NECTIN2 NMT1 NOL3 OXA1L PARK7 PARP1 PEMT PET100 PEX5 PHB2 PLA2G6 POLDIP2 POLG PPARGC1B PRDX3 PRELID1 PSMB7 PSMD8 PTCD2 PTRH1 PUS1 RAB29 RAB3A RAC2 SAE1 SDHAF1 SEPT4 SFN SHARPIN SIRT3 SLC25A4 SLC35F6 SQSTM1 STARD7 STAT3 STOML2 TFB1M TFDP1 TIMM13 TIMM17B TIMM22 TIMM44 TIMM50 TIMM8B TMEM102 TOMM22 TOMM34 TOMM40 TOMM7 TP53 TRMU TSPO UBE2J2 UBL5 UQCC2 UQCC3 UQCR10 USP36 VAT1 VDAC1 VPS11 WDR45 WIPI1 WIPI2 ZNF205* |
| **GO:0006412~translation (*overrepresented among DEGs with lower expression in the LRFI group vs HRFI)*** |
| *AARS AARS2 ABCF1 AGO1 AIMP2 ATF4 BOLL CALR CARS CCL5 CDK5RAP1 CHCHD1 CNOT3 CNOT9 COA3 CTIF DALRD3 DHPS DTD1 EARS2 EEF1B2 EEF1G EEF2 EEF2K EFTUD2 EIF1 EIF2AK1 EIF2B1 EIF2B2 EIF2B3 EIF2B4 EIF2B5 EIF2D EIF3D EIF3G EIF3I EIF3K EIF3L EIF4A1 EIF4A3 EIF4E2 EIF4EBP1 EIF4EBP2 EIF4EBP3 EIF4G1 EIF5A ELAVL1 ERBB2 FARSA FXR2 GADD45GIP1 GAPDH GARS GCN1 GTPBP1 HARS IGHMBP2 ILF3 IMP3 KARS KLHL25 LARP1 MAPK1 MEX3D MPV17L2 MRPL10 MRPL11 MRPL14 MRPL15 MRPL16 MRPL17 MRPL18 MRPL2 MRPL20 MRPL21 MRPL24 MRPL28 MRPL36 MRPL38 MRPL4 MRPL40 MRPL43 MRPL44 MRPL46 MRPL48 MRPL49 MRPL52 MRPL54 MRPS11 MRPS14 MRPS2 MRPS24 MRPS25 MRPS26 MRPS27 MRPS34 MRPS6 MRPS7 MRRF MRTO4 MTERF4 MTOR NHP2 PARS2 PCBP2 PDF POLDIP3 POLR2G PRKRA PTRH1 PYM1 RAN RARA RPL11 RPL12 RPL13 RPL15 RPL18A RPL19 RPL27 RPL29 RPL3 RPL30 RPL35 RPL36 RPL38 RPS11 RPS13 RPS15 RPS18 RPS19 RPS23 RPS28 RPS3 RPS5 RPS6KA1 RPS6KB2 RPS7 RPS8 RPS9 SLC25A39 SARS SLC25A1 SLC25A11 SLC25A12 SLC25A19 SLC25A2 SLC25A20 SLC25A23 SLC25A26 SLC25A28 SLC25A3 SLC25A34 SLC25A38 SLC25A4 SLC25A44 SLC25A47 SNU13 STAT3 TARBP2 TNIP1 TRAP1 UBA52 UCP2 UQCC2 WARS YARS* |
| **GO:0006091~generation of precursor metabolites and energy (*overrepresented among DEGs with lower expression in the LRFI group vs HRFI)*** |
| *ACO2 ACOX1 AKT2 ATP5F1B ATP5F1D ATP5IF1 BLOC1S1 BNIP3 C1QTNF2 CAVIN3 CBFA2T3 CHCHD10 COQ9 COX17 COX3 COX4I1 COX5A COX6A1 COX6A2 COX7A1 CS DLST ENO1 ENO3 ETFB FDXR FECH FH FXN GADD45GIP1 GALK1 GAPDH GCK GNPDA1 GPD1 GPI GRB10 GYS1 HK1 IDH2 IDH3B IDH3G ME3 MECP2 MTFR1L MTOR MYBBP1A MYOG NDUFA1 NDUFA10 NDUFA13 NDUFA4 NDUFA8 NDUFAB1 NDUFB1 NDUFB11 NDUFB2 NDUFB6 NDUFB7 NDUFB9 NDUFS2 NDUFS3 NDUFS5 NDUFS6 NDUFS7 NDUFV1 NDUFV2 OGDH OXA1L PARK7 PCDH12 PFKFB1 PFKFB4 PFKL PFKM PFKP PGAM1 PGAM2 PGM1 PGM5 PHKG1 PKM PPARD PPP1R3F PRELID1 PRKAG1 PRKAG3 PYGL PYGM SDHAF2 SDHB SDHC SIRT3 SIRT6 SLC25A12 SLC25A23 SLC25A3 SLC25A4 SLC37A4 STAT3 SUCLG1 THTPA TRAP1 UBA52 UBB UBC UQCC2 UQCC3 UQCR10 UQCRC1 UQCRFS1 UQCRQ* |
| **GO:0055114~oxidation-reduction process (*overrepresented among DEGs with lower expression in the LRFI group vs HRFI)*** |
| *ABCD1 ACACB ACAD8 ACAD9 ACADS ACAT2 ACO2 ACOT8 ACOX1 ACOX3 ACSS2 ADI1 ADIPOR1 ADIPOR2 AIFM1 AIFM2 AKR1B1 AKT2 ALDH16A1 ALDH3A2 ALDH4A1 ALDH9A1 ALKBH3 ALKBH6 ALKBH7 AMACR AOC3 BLOC1S1 BLVRA BLVRB BNIP3 C1QTNF2 CAVIN3 CBFA2T3 CHCHD4 COQ6 COQ9 COX3 COX4I1 COX5A COX6A1 COX6A2 CPT2 CRAT CRYL1 CRYM CS CYB5B CYB5R1 CYB5R3 CYGB DHCR24 DHCR7 DHDH DHRS1 DHRS11 DHRS3 DHRS7C DLST DOHH DUS2 ECH1 ECHDC2 ECI1 ECI2 ECSIT EGLN2 ENO1 ENO3 ETFB FAM213B FDFT1 FDXR FH FOXRED1 FXN GAPDH GCK GDI1 GDI2 GFER GFOD1 GLRX3 GLRX5 GLYR1 GMPR GPD1 GPD1L GPI GRB10 GRHPR GSTK1 GYS1 HADH HADHA HCCS HIBADH HK1 HMOX2 HPDL HR HSD17B10 HSD17B14 HSD17B8 HSD3B7 IDH2 IDH3B IDH3G IL4I1 IMPDH1 IMPDH2 IVD JMJD6 KDM4B KDM8 LDHD LOXL4 MAOB MAPK14 MARC1 MARC2 ME3 MECP2 MECR MICAL3 MMACHC MSRA MTFR1L MTHFD1 MTHFR MTOR MYBBP1A NDUFA1 NDUFA10 NDUFA13 NDUFA4 NDUFA8 NDUFAB1 NDUFB1 NDUFB11 NDUFB2 NDUFB6 NDUFB7 NDUFB9 NDUFS2 NDUFS3 NDUFS5 NDUFS6 NDUFS7 NDUFV1 NDUFV2 NQO2 NSDHL NXN OGDH OGFOD2 OGFOD3 OXA1L P3H3 P4HTM PARK7 PCBD1 PCDH12 PCK2 PEX5 PFKFB1 PFKFB4 PFKL PFKM PFKP PGAM1 PGAM2 PGM1 PGM5 PHF2 PHKG1 PIR PKM PLIN5 PLOD1 PNPO PPARD PPP1R3F PRDX1 PRDX2 PRDX3 PRDX4 PRDX5 PRDX6 PRELID1 PRKAG3 PTGES2 PYGL PYGM QDPR QSOX1 SCD SDHAF2 SDHB SDHC SDR39U1 SIRT3 SLC25A12 SLC37A4 SMOX SOD1 SOD3 SORD SPR STEAP3 SUCLG1 TM7SF2 TRAP1 TXN2 TXNDC12 TXNDC17 TXNRD2 TXNRD3 TYSND1 UBA52 UBB UBC UQCC3 UQCR10 UQCRC1 UQCRFS1 UQCRQ VAT1* |
