# Supplementary material for: A comparative analysis of the transcriptome profiles of liver and muscle tissue in pigs divergent for feed efficiency
Source: BMC Genomics. 2019 Jun 6;20:461. doi: 10.1186/s12864-019-5740-z (PMC6555042; doi:10.1186/s12864-019-5740-z)
Supplement: Supplementary file 6 — Table S6. Selected gene ontology terms in liver. This table lists the genes involved in a selected number of gene ontology terms identified in liver. (DOCX 16 kb) [file 12864_2019_5740_MOESM6_ESM.docx]

| ***GO:0006952~defense response (overrepresented among DEGs with higher expression in the LRFI group vs HRFI)*** |
| --- |
| *ABCF3 ADAR APOA4 ATP6V1H C1QA C1QC CASP1 CD5L CNR2 CRP CSF1R CUL1 CXCL10 CYBB DAPK1 DDX3X DHX58 DNAJA3 F3 FOS HAVCR2 HERC5 HYAL2 IFI27 IFI44L IFIT1 IFIT2 IFIT3 IFIT5 IFNGR1 IKBKB IL17B IL1RAP IRF9 ITIH4 JAK1 LAMP1 LEAP2 MARCO MVK MX1 MX2 MYC NCF2 NLRP3 NR1D2 OAS1 OASL OSMR PARP9 PLGRKT PML POLR3E PPARA PSME4 RABGEF1 RNF216 RORA RSAD2 SBNO2 SGMS1 SP140 TCIRG1 TRAF6 TRIM21 XAF1 ZBP1* |
| ***GO:0034097~response to cytokine (overrepresented among DEGs with higher expression in the LRFI group vs HRFI)*** |
| *ADAR ADIPOR2 CD274 CMKLR1 CSF1R CXCL10 DAPK1 DNAJA3 F3 FGF23 FOS HYAL2 IFI27 IFIT1 IFIT2 IFIT3 IFNGR1 IKBKB IL1RAP IRF9 ITIH4 JAK1 JUN MCL1 MX1 MX2 MYC NFIL3 NPNT NPR2 OAS1 OASL OSMR PARP9 PML PRPF8 PSME4 RABGEF1 RORA RSAD2 SBNO2 SGMS1 TRAF6 TRIM21 XAF1* |
| ***GO:0060337~type I interferon signalling pathway (overrepresented among DEGs with higher expression in the LRFI group vs HRFI)*** |
| *ADAR IFI27 IFIT1 IFIT2 IFIT3 IRF9 JAK1 MX1 MX2 OAS1 OASL RSAD2 XAF1* |
| ***GO:0022613~ribonucleoprotein complex biogenesis (overrepresented among DEGs with higher expression in the LRFI group vs HRFI)*** |
| *ADAR AGO2 ATXN2L BYSL C1D CLP1 DDX28 DDX39B DDX3X DHX37 EXOSC1 EXOSC2 FTSJ3 GEMIN5 GNL3 IMP3 MRM3 MRPL1 NOB1 NOCT NOL6 NUFIP1 PATL1 PRPF8 RIOK2 RPL7L1 RRP9 RRS1 SF1 STRAP UTP3 WDR43 WDR74 XAB2 YAE1D1* |
| ***GO:0055088~lipid homeostasis (overrepresented among DEGs with higher expression in the LRFI group vs HRFI)*** |
| *ABCA1 ABCG1 ACAD11 ACADL ACOX1 ALMS1 APOA4 APOA5 ETFA LIPG NPC1 NR1D2 NR5A2 RORA* |
| ***GO:0007166~cell surface receptor signalling pathway (overrepresented among DEGs with lower expression in the LRFI group vs HRFI)*** |
| *ADAM33 ADAM8 ADGRG5 AGO4 ANGPT4 ANXA4 ASPN BEX3 BIRC6 BMP6 CAPRIN2 CBLB CCBE1 CCL21 CCNY CD19 CD27 CD3D CD79A CD79B CD8A CHRDL1 CIITA CITED2 CYFIP2 DAB2IP DCN DKK3 DMTN DTX1 EDA EFEMP1 EPHA7 EPHB2 FAF1 FCER1A FLT3 FMOD FYN FZD10 GAS6 GMDS GNG2 GPC1 GRB7 HEYL HOMER3 HRG HTRA1 HTRA3 IGFBP6 IL17RD IL2RG ITGA3 ITGB7 ITGBL1 ITM2C KIAA0141 KLHL6 KRT19 LIMK1 LPXN LRP6 LRRK2 LTBP2 LTF MAP3K1 MATK MFNG MMP2 MMP9 MS4A2 MTMR4 NOV NPY1R OGN PAK1 PDGFA PLP1 PLP2 PLPP4 PMEPA1 PTK7 PTPRD PYGO1 RAB7A RARG RPS19 SEMA3G SERPINE2 SFRP1 SFRP5 SH2D2A SKAP1 SLA-DRA SLIT3 SORT1 SOX4 SOX9 STK36 SULF1 TBX20 TNF TSPAN17 UNC13B UPK1B VIL1 VIM WIPF2 WNT5A WNT9A ZNF703* |
| ***GO:2000026~regulation of multicellular organismal development (overrepresented among DEGs with lower expression in the LRFI group vs HRFI)*** |
| *ADAM8 ADM2 ANGPT4 AQP3 ARHGDIB ASPN BMP6 CAPRIN2 CCBE1 CCSAP CD27 CD34 CITED2 CLIP1 DAB1 DAB2IP DCN DTX1 EAF2 EFEMP1 EPHA7 EPHB2 FMOD FYN GAS2L1 GAS6 GPC1 HEYL HRG IL13 ITGA3 ITM2C LAMA4 LGALS1 LIMK1 LRP6 LRRK2 LTF MEOX2 MGP OGN OMD PDGFA PTGIS PTK7 PTPRD RARG RASAL1 RBP1 SASH3 SEMA3G SERPINE2 SFRP1 SFRP5 SLA-DOA SLIT3 SOX9 SSH3 SULF1 TBX20 TESC TFRC TIMP1 TNF TRPV2 VIM WNT5A WNT9A WT1 ZNF703* |
| ***GO:0040011~locomotion (overrepresented among DEGs with lower expression in the LRFI group vs HRFI)*** |
| *ADAM8 ANGPT4 ARHGDIB ATP8A1 CCBE1 CCL21 CCSAP CD34 CFAP20 CITED2 CNN2 DAB1 DAB2IP DCN DMTN DOCK7 EPHA7 EPHB2 EPX EZR FAP FMOD FSCN2 FYN GAS6 GPC1 GRB7 HRG IL16 ITGA3 ITGB7 LAMA4 LRRK2 LYST MATK MATN2 MEOX2 MMP9 MYO5A NET1 NOTCH1 NOV OGN PAK1 PDGFA PDPN PLP2 PTK7 PTPRO RAC2 RIPK3 RPS19 S100A14 SEMA3C SEMA3D SEMA3G SERPINE2 SFRP1 SLIT3 SOX9 SPTAN1 SULF1 TBX20 TIMP1 TNF TRPM2 VIL1 WNT5A ZNF268 ZNF703* |
